# P-1467. Clinical Development of MenABCWY (PenbrayaTM), a Broadly Protective Pentavalent Meningococcal Vaccine

**DOI:** 10.1093/ofid/ofaf695.1653

**Published:** 2026-01-11

**Authors:** Lefteris Zolotas, Ashlesh Murthy, Roger Maansson, Ryan Newton, Kelly Belanger, Yanping Liu, Robert O’Neill, Mark W Cutler, Annaliesa S Anderson, Johannes Beeslaar

**Affiliations:** Pfizer Ltd, Marlow, England, United Kingdom; Pfizer Inc, Pearl River, New York; Pfizer Vaccine Clinical Research and Development, Collegeville PA, Collegeville, PA; Vaccine Research and Development, Pfizer Ltd, Marlow, England, United Kingdom; Pfizer, Pearl River, NY; Pfizer Inc, Pearl River, New York; Pfizer Inc, Pearl River, New York; Pfizer, Inc. Pearl River, NY, Pearl River, NY; Pfizer, Pearl River, NY; Pfizer Vaccine Clinical Research and Development, Hurley, Berkshire UK, Hurley, Berkshire, England, United Kingdom

## Abstract

**Background:**

The first-in-class pentavalent meningococcal serogroup A/B/C/W/Y vaccine (MenABCWY; Penbraya^TM^) received US licensure in 2023 as a 2-dose, 0,6-month (m) series for individuals 10‒25 years (y) of age. Here we review data supporting MenABCWY licensure and utility.

**Figure:**
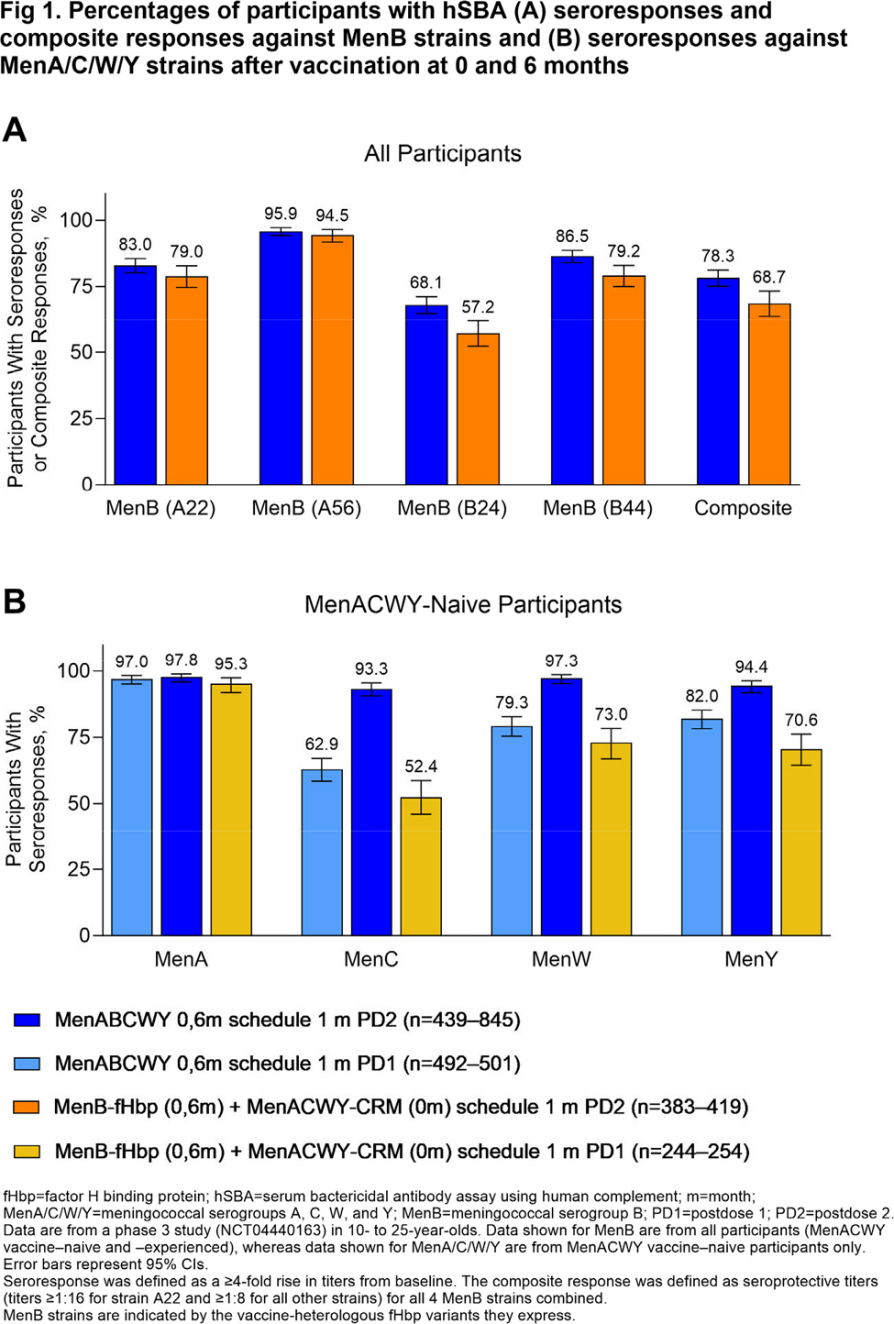

**Figure:**
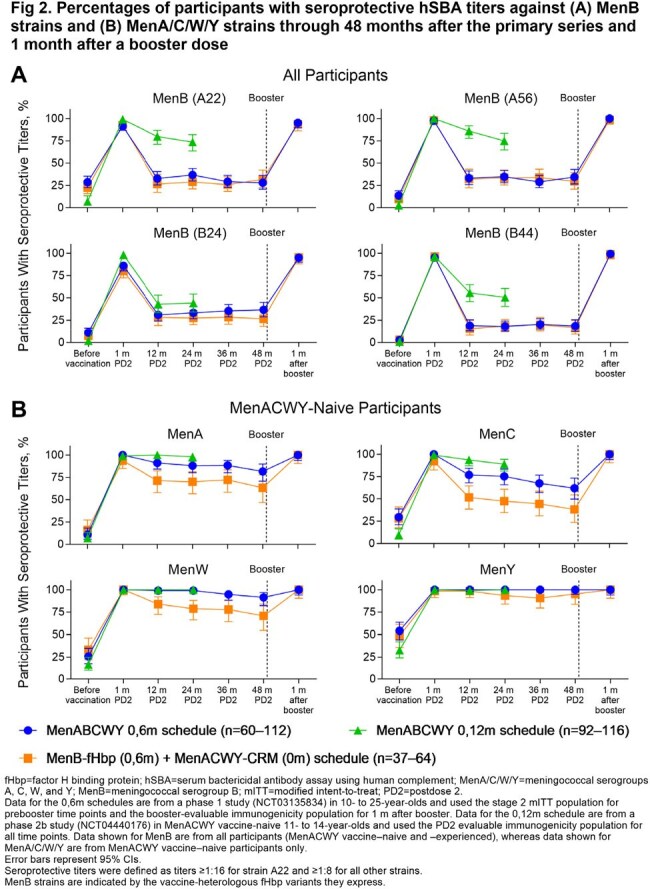

**Methods:**

Three phase 1/2b/3 MenABCWY studies included a total of > 4000 MenB vaccine–naive, MenACWY vaccine–naive or –experienced participants 10‒25 y of age. Immune responses were assessed as serum bactericidal antibody using human complement (hSBA) against MenA/C/W/Y strains and 4 diverse, vaccine-heterologous MenB strains; endpoints included seroprotection (titers ≥ 1:8 or ≥ 1:16, depending on strain) and seroresponse (≥ 4-fold titer rise from baseline) rates and geometric mean titers (GMTs). Safety was also assessed.

**Figure:**
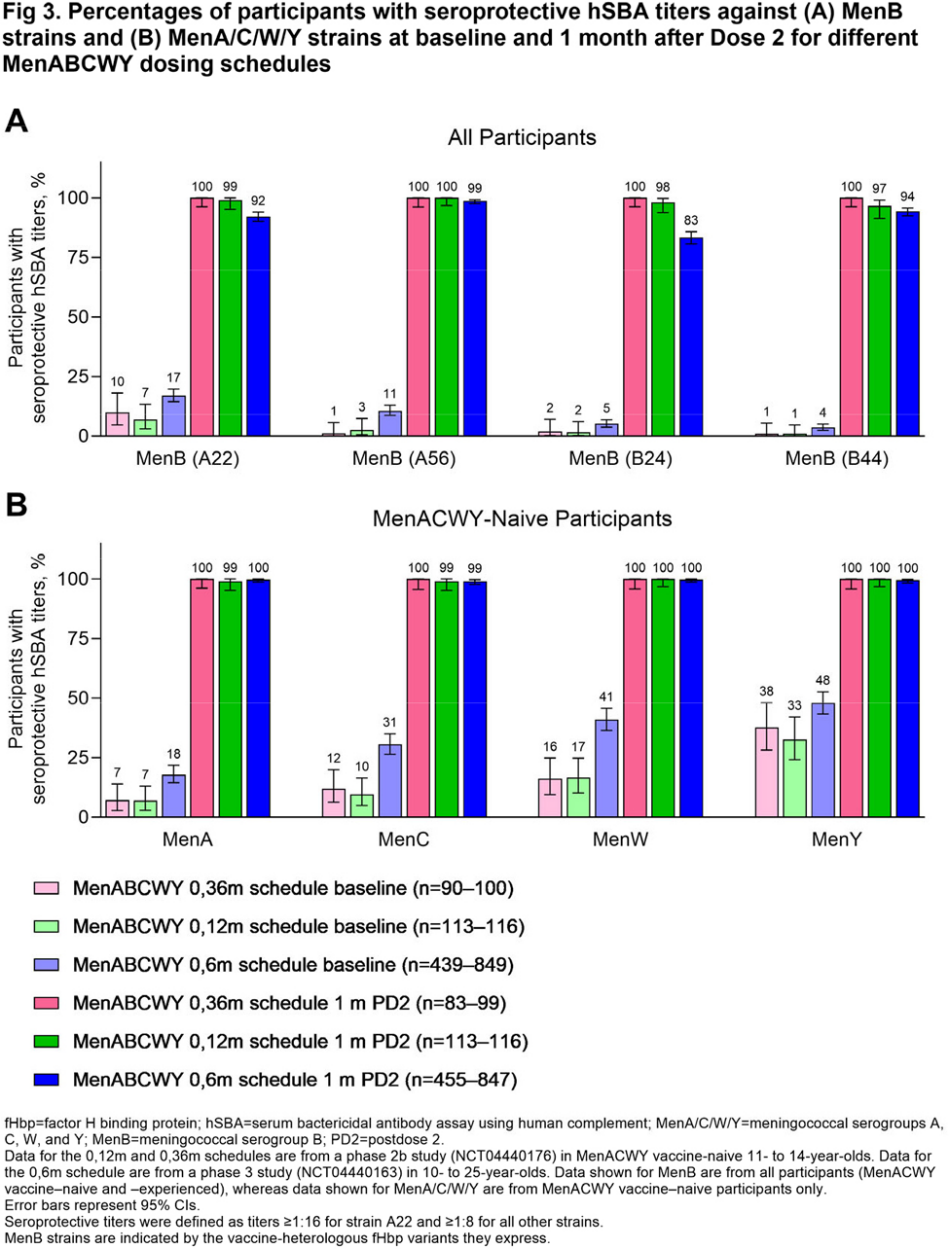

**Figure:**
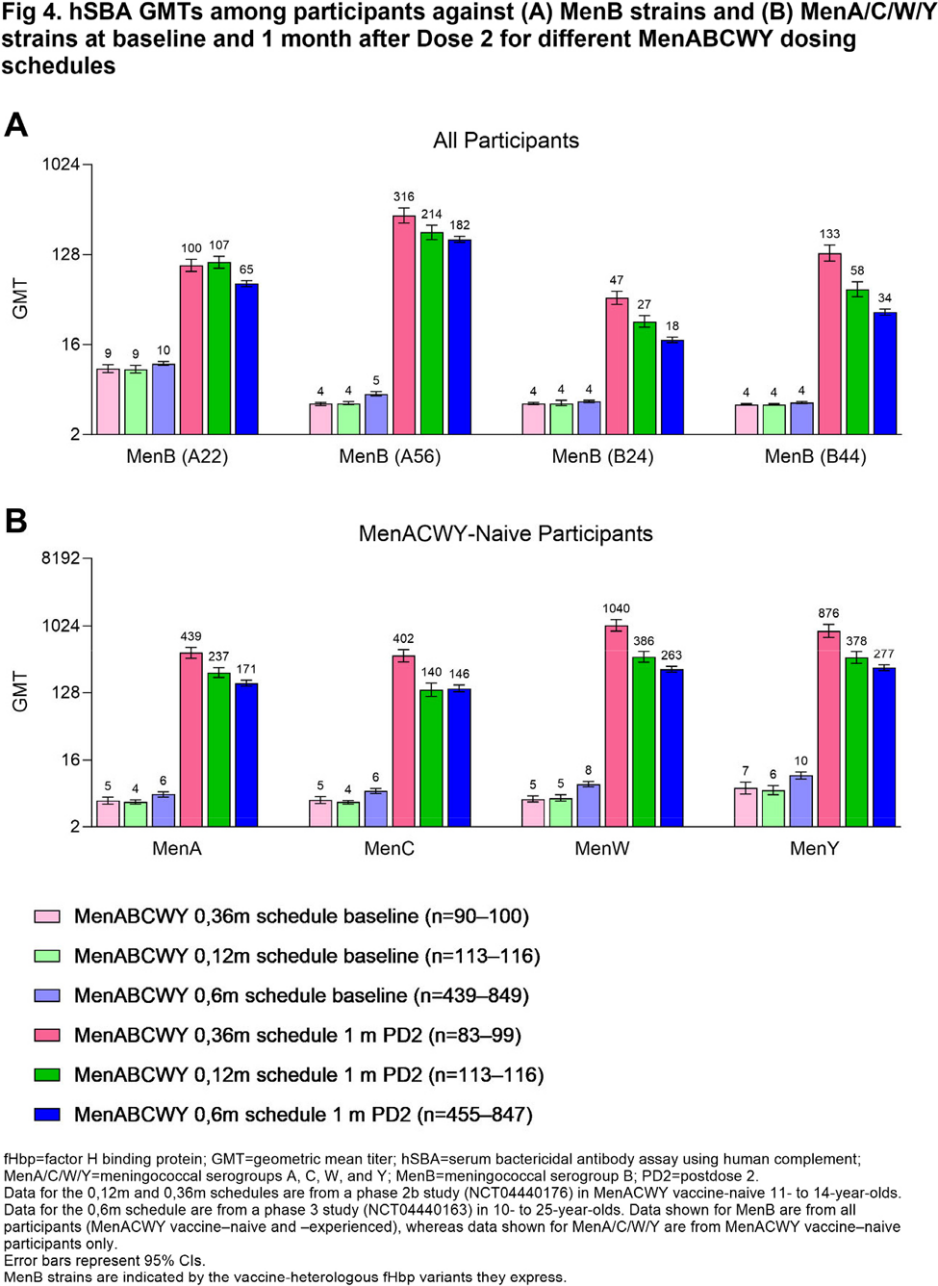

**Results:**

MenB hSBA seroresponse (68.1%‒95.9%) and composite response rates (78.3%; seroprotection for all 4 strains) after Dose 2 of a 0,6m MenABCWY series were similar to or higher than after 2 MenB-fHbp doses (Fig 1A), achieving noninferiority at the −10% margin. Results were similar for MenA/C/W/Y seroresponse rates after each MenABCWY dose among MenACWY-naive (Dose 1, 62.9%‒97.0%; Dose 2, 93.3%‒97.8%; Fig 1B) and -experienced participants compared with after 1 MenACWY-CRM dose. Seroprotection rates after a 0,6m MenABCWY series were similar to or higher than for MenACWY-CRM+MenB-fHbp through 4 y and indicated anamnestic responses to a MenABCWY booster at 4 y (MenB, ≥ 95.1%; MenA/C/W/Y, 100%; Fig 2). Extended 12-m and 36-m MenABCWY dosing intervals led to ≥ 96.6% MenA/B/C/W/Y seroprotection rates (Fig 3); GMTs (Fig 4) and seroresponse rates generally trended higher as the interval increased from 6 to 36 m. Seroprotection rates through 24 m after the 0,12m series were similar to or higher than for the 0,6m series (Fig 2). MenABCWY was well tolerated; there were no safety concerns.

**Conclusion:**

MenABCWY is well tolerated with an acceptable safety profile. A 0,6m MenABCWY series induces robust immune responses that are similar to or higher than for separate MenACWY and MenB vaccines through 4 y, boostable, and remain similar or increase with longer dosing intervals up to 36 m. These results suggest comprehensive protection and flexibility for meningococcal vaccination using a single vaccine. Funded by Pfizer.

**Disclosures:**

Lefteris Zolotas, MD, Pfizer Inc: Employee Ashlesh Murthy, MD, Pfizer Inc: Employee|Pfizer Inc: Stocks/Bonds (Public Company) Roger Maansson, MS, Pfizer Inc: Employee Ryan Newton, BSC/MBA, Pfizer Inc: Employee Kelly Belanger, BS, Pfizer Inc: Employee Yanping Liu, PhD, Pfizer Inc: Employee Robert O’Neill, PhD, Pfizer Inc: Employee Mark W. Cutler, PhD, Pfizer Inc: Employee Annaliesa S. Anderson, PhD, Pfizer Inc: Employee Johannes Beeslaar, MD, Pfizer Inc: Employee

